# Genome Sequencing of Blacklip and Greenlip Abalone for Development and Validation of a SNP Based Genotyping Tool

**DOI:** 10.3389/fgene.2018.00687

**Published:** 2019-01-04

**Authors:** James Kijas, Matthew Hamilton, Natasha Botwright, Harry King, Luke McPherson, Anton Krsinich, Sean McWilliam

**Affiliations:** ^1^CSIRO Agriculture and Food, Queensland Bioscience Precinct, Brisbane, QLD, Australia; ^2^CSIRO Agriculture and Food, Hobart, TAS, Australia; ^3^Craig Mostyn Group, Jade Tiger Abalone, Indented Head, VIC, Australia

**Keywords:** abalone, SNP, whole genome sequence, genotyping tool, genetic diversity

## Abstract

Abalone breeding in southern Australia often involves the production of interspecies hybrids through crossing blacklip (*Haliotos rubra*) and greenlip (*H. laevigata*) parental populations. To assist applied breeding and investigate genetic divergence, this study applied genome sequencing and variant detection to develop and validate a SNP genotyping tool. Skim short read Illumina sequencing was performed using 24 individuals from each of the two parental species and a hybrid population. Raw reads were assembled into three population specific pools (each 12–15 fold coverage), before mapping was performed against a draft greenlip abalone reference genome. Variant detection identified 22.4 M raw variants across the three populations (SNP and indels), suggesting they are highly heterozygous. First stage filtering defined a high quality SNP collection of 2.2 M variants independently called in each of the three populations. Second stage filtering identified a much smaller set of variants for assay design and genotyping using a validation set of 191 abalone of known population and pedigree. Comparison of allele frequency data revealed a high proportion of SNP (43%) had divergent allele frequency (< 0.2) between the two parental populations, suggesting they should have utility for parentage assignment. A maximum likelihood approach was used to successfully assign 105 of 105 progeny to their known true parent amongst a set of 86 candidate parents, confirming the genotyping tool has utility for applied breeding. Analysis of pairwise allele sharing successfully discriminated animals into populations, and PCA of genetic distance grouped the hybrid animals with intermediate values between the two parental populations. The findings present a library of DNA polymorphism of utility to breeding and ecological application, and begins to characterize the divergence separating two economically important aquaculture species.

## Introduction

The acquisition of genetic data is essential to modern aquaculture breeding programs for the assignment of pedigree prior to estimation of broodstock breeding values. Genetic information facilitates communal rearing during progeny testing to reduce environmental variation, rectify pedigree errors which if undetected negatively affect genetic gain (McClure et al., 2018) and represents the raw material required to monitor and manage inbreeding (Atin et al., 2017). Each of these applications can be achieved using a modest number of loci (< 100), however, more advanced genomic resources are required to perform genome wide association studies to explore the genetic basis of complex traits and implement genomic prediction. This is well underway in a number of aquaculture species (reviewed by Aquaculture Genomics et al., 2017) however, mollusc genomes pose a number of challenges that includes high nucleotide diversity and segregation distortion (Hollenbeck and Johnston, 2018). This, and the comparatively small scale of farmed abalone production, likely explains why genomic tool development for abalone (genus *Haliotis*) has lagged behind other species. This is changing with development of first draft reference genome assemblies for both pacific (*H. discuss hannai*; Nam et al., 2017) and greenlip abalone (*H. laevigata*; Botwright et al., unpublished). Further, generation of *de novo* transcriptomes (*H. tuberculate*; Harney et al., 2016, *H. midae*; Bester-Van Der Merwe et al., 2013) and construction of large insert libraries (*H. diversicolor*; Jiang et al., 2016) is starting to provide the tools required for better management of breeding programs and the ability to investigate the biological basis of key traits.

Australia is home to six temperate species of abalone which are genetically distinct from *Haliotis* species endemic to New Zealand, Indonesia, Thailand, India and beyond (Degnan et al., 2006). The Australian abalone farming industry is primarily based on two of these, the blacklip (*H. rubra*) and greenlip (*H. laevigate*) abalone which are often hybridized to generate an interspecies offspring favored for its improved growth and production. At Jade Tiger Abalone a family based selective breeding program has been in operation since 2003, aiming to maintain diverse populations of both parental species (blacklip and greenlip) and produce hybrids selected for growth, meat yield and product quality (Hamilton et al., 2009). The reproductive biology of abalone makes the task of tracking parentage a challenge, as they are broadcast spawners where fertilization occurs outside the animal. Further, in the absence of sophisticated approaches to condition and induce gamete release by individual broodstock, they are social or group spawners. Infrastructure design alone is often not a practical solution for the generation and rearing of progeny with known parentage, meaning the application of DNA data is important. Family assignment has been performed in the Jade Tiger Abalone breeding program using a panel of microsatellites, however, the opportunity to more fully exploit the power of advanced genomics prompted the main objectives of this study. We sought to (i) develop a library of single nucleotide polymorphisms (SNP) suitable for future genomic tool development; (ii) design and test a low density genotyping tool capable to assigning pedigree, and (iii) commence characterization the genetic divergence separating the two abalone species of primary importance to the abalone farming in Australia.

## Materials and Methods

### Whole Genome Sequencing, SNP Discovery and Development of a Genotyping Tool

A total of 24 animals were obtained from the 2012 year class managed by Jade Tiger Abalone at Indented Head in Victoria, Australia (Hamilton et al., 2009). Pedigree data was used to sample eight blacklip (BL), eight greenlip (GL), and eight hybrid (HY) animals to maximize diversity and minimize relatedness. Genomic DNA was extracted using a modified CTAB method (Botwright et al., unpublished) with quality visually assessed on 1.2% agarose gel and quantity determined using a NanoDrop spectrophotometer (Thermo Scientific). DNA was used for Nextera DNA sample library construction (Illumina) and 100 base pair paired-end sequencing using the Illumina HiSeq 2000 (Australian Genome Research Facility, Melbourne, Australia). Raw sequence reads were quality trimmed using quadtrim^1^ with options -q 20 -a 20 -l 50 -p 3. Trimmed sequences were aligned to the greenlip abalone draft reference assembly (Botwright et al., unpublished) using BWA-MEM (Li and Durbin, 2009) with default parameters. Due to the large number of contigs in the current genome assembly (> 100,000) only sequences mapping to contigs > 100 kb that had an annotated known gene were used for subsequent variant detection. BAM files were processed with GATK to include indel realignment and duplicate removal (McKenna et al., 2010). SNP and INDEL discovery was undertaken using the GATK haplotype caller on individual samples followed by combined genotyping with GATK genotypegvcfs on population pools according to GATK best practice (Van der Auwera et al., 2013). Variants were filtered using bcftools^2^ to retain SNP with read depth of at least 10 and a minor allele frequency between 0.25 and 0.75 in every pool. Additional filters were applied to remove SNP with flanking variants within 50bp and within 1kb of scaffold ends in preparation for genotyping tool design. A total of 384 prioritized SNP loci were submitted to the Center for Aquaculture Technologies (San Diego, California) for assay design for genotyping using the kompetitive allele-specific PCR (KASP) technology of LGC genomics (Middlesex, United Kingdom).

### Validation Populations and SNP Data Filters

The population used for validation consisted of 191 animals collected from the Jade Tiger Abalone breeding program. This included 65 blacklip abalone comprising 33 broodstock (year class 2012–2014) and 32 offspring. The BL offspring were generated using two known dams and unknown sire due to the design of the breeding program (Hamilton et al., 2009). The validation set also included 87 greenlip abalone comprising 53 broodstock (year class 2013 and 2014) and 34 offspring. The GL offspring were generated using unknown dams and two known sires. The remaining 39 animals are BL × GL hybrids generated using unknown dams and the same two GL sires used to construct the GL progeny. Each known broodstock parent is present in the validation population, and the pedigree is recorded in the PED and MAP files formatted for analysis in PLINK v1.9 (Chang et al., 2015). Extracted genomic DNA was submitted to the Center for Aquaculture Technologies (San Diego, CA, United States) for KASP genotyping as a commercial service. Data was returned for 384 loci before PLINK v1.9 was used to sequentially apply four quality filters prior to additional analysis (Supplementary Table S1). First, 179 SNP (or 47%) were removed with call rate < 0.9 across the set of 213 animals. Of these, 93 (or 25%) failed in all samples and a further 86 (or 22%) had a non-zero call rate < 0.9. Second, 88 monomorphic SNP were removed. Third, population specific genotype frequencies were estimated and 12 SNP removed which were heterozygous in all animals within at least one population. Finally, 22 animals were removed which had call rate < 0.90 within the set of 105 SNP remaining after the previous steps. The final dataset contained 191 animals and 105 loci (Supplementary Table S1).

### Genetic Diversity, Distance and Parentage Assignment

Estimates of genetic diversity, including the proportion of polymorphic loci (*P*_N_), expected heterozygosity (*H*_E_), and allele frequency were calculated using PLINK v1.9. The relative genetic distance between animals was assessed using two methods. First, principal component analysis (PCA) of pairwise genetic distance was estimated as implemented in PLINK v1.9 using the –pca flag. Second, pairwise allele sharing (*A*_S_) was estimated as an identity by state matrix using the – distance ibs flag. The *A*_S_ matrix was used to generate a heatmap and unsupervised dendogram using R software package RColorBrewer. The ability of the SNP genotyping tool to accurately estimate parentage was evaluated using Cervus v 3.0.7 (Kalinowski et al., 2007), which is written and maintained by Tristan Marshall (Field Genetics, London, United Kingdom). The software implements a likelihood based approach to assign parent – progeny pairs using all available genotypes. The identity of 36 candidate sires and 50 candidate dams was provided which included the known true parents. The identifiers of 105 progeny was also supplied, before two key metrics were estimated for each. The first is a log transformed likelihood ratio for every candidate parent (*LOD*, see Kalinowski et al., 2007). The second is *deltaLOD*, which gives the difference in *LOD* score between the most likely candidate parent of a given progeny and the second most likely candidate parent. Increasingly positive *deltaLOD* values reflect elevated confidence that the best candidate parent is the true parent where all putative parents have been genotyped. The candidate parent with the highest *deltaLOD* was compared with the known parent to establish the true parent assignment rate.

### Data Availability Statement

The raw sequence and variant files containing the SNP collections have been made available via the publically available CSIRO Data Access Portal. It can be obtained using doi: https://doi.org/10.4225/08/5b0cf4a0bfd9b Additional data supporting the conclusions of this manuscript will be made available by the authors, without undue reservation, to any qualified researcher.

## Results

### Genome Sequencing for Variant Detection

To commence the development of SNP collections, DNA samples from blacklip (BL), greenlip (GL) and BL × GL hybrid abalone (HY) were used for Illumina short read skim sequencing. This returned a total of 58.88 Gb of raw data, however, the average depth of coverage per individual was approximately 2.5 fold prompting us to pool sequence reads within population prior to variant discovery. This generated three pools (BL, GL, HY), each with 12 – 15 fold genome coverage which is considered more than adequate for variant detection (Nielsen et al., 2011). Reads derived from greenlip abalone mapped with the highest success rate to the reference assembly (98.1%, Supplementary Table S2). The success rate was only slightly reduced for the blacklip (97.2%) and hybrid abalone sequences (97.7%). Mapping efficiency was also high for reads mapped in the correct orientation and spacing relative to their mate pair, suggesting a high quality dataset suitable for variant detection (84%, Supplementary Table S2). A total of 22.4 M raw variants were identified and used for filtering based on sequence quality, read depth and missingness acros pools. A total of 156,000 high quality variants were identified, of which approximately 44% were independently identified as polymorphic in both the BL and GL pools. The observation that around half the identified variants in one species were independently observed in the other strongly suggests low evolutionary divergence separates the species, consistent with the observation the two species are capable of producing fertile interspecies hybrids.

### SNP Tool Development

To drive selective breeding, a key objective was to develop a low density SNP genotyping tool suitable for parentage assignment. In response to the commercial preference for BL × GL hybrids, the genotyping tool was enriched with SNP displaying polymorphism within each of the two parental species and hybrid populations. The pooled NGS approach used for SNP discovery generated an estimate of population minor allele frequency for each of the 2.9 M high quality variants. This was used to identify a subset of 156,000 SNP with allele frequency between 0.25 and 0.75 in all three pools. To prioritize loci further, additional filters were applied that considered the proximity of surrounding variants and their location on the reference genome assembly (Materials and Methods). A total of 384 prioritized loci were subjected to KASP assay design before the tool was assessed by genotyping a validation set of 213 abalone. Preliminary assessment revealed nearly a quarter of assays (93/384) returned no genotypic data due to failed synthesis and a further quarter (88/384) reported monomorphic results across all animals (Supplementary Table S1). Following additional filtering, the final dataset comprised 105 loci and 191 animals. The proportion of validated assays was low (27% or 105/384) and it is unclear if this reflects undetected genome complexity that disrupted the assays, a technical failure of KASP, the variant discovery approach undertaken or some combination.

### Genetic Diversity Within Populations

**FIGURE 1:**
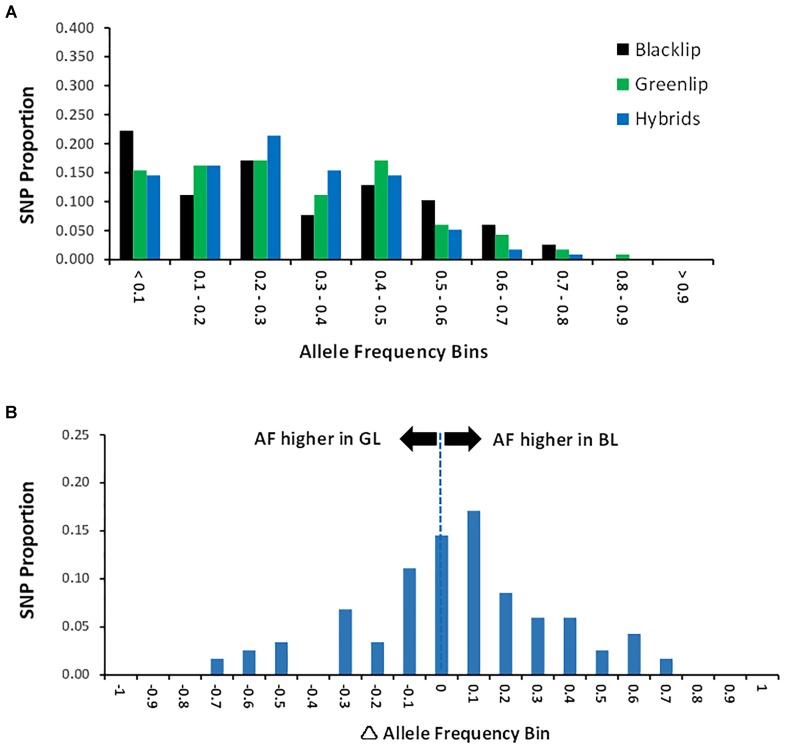
Allele Frequency. **(A)** Allele frequency (AF) was estimated jointly across the three populations and the proportion of SNP is shown across 10 frequency bins. AF is higher than 0.5 when the minor allele in one population is the major allele in a separate population. **(B)** Allele frequency difference (AFΔ) was estimated for each SNP as the BL AF minus the GL AF. Positive AFΔ values indicate SNP with higher allele frequency in BL compared with GL. The distribution of values is given in frequency bins.

To commence analysis of genetic diversity, four metrics were estimated and compared between populations (Table 1). The proportion of polymorphic loci observed was higher in both the BL and GL samples when compared to the hybrids. Similarly, the average allele sharing was lower within both BL and GL compared to the hybrids which were more similar to each other (higher average *A*_S_). This may seem counter intuitive, however, it likely reflects the presence of diverse broodstock in the BL and GL populations not present in the hybrids. Further, fewer hybrids were genotyped and all were generated in matings involving only two greenlip sires (Materials and Methods). While a slightly lower proportion of SNP were polymorphic in the hybrids, they displayed an enrichment of SNP with high allele frequency (Figure 1). Specifically, the hybrids had an elevated proportion of SNP in AF range from 0.1 to 0.4. Conversely, the parental species displayed an excess of SNP with low AF (either < 0.1 or > 0.5; Figure 1). To continue exploration of patterns of diversity, we next assessed the difference in allele frequency (AFΔ) by comparing blacklip and greenlip (Figure 1). This revealed blacklip had generally higher AF (and positive AFΔ values). Importantly, a high proportion of loci (44% or 44/105) had values greater than ± 0.2 which suggests the genotyping tool is likely to differentiate between BL and GL and their hybrids in parentage assignment.

**Table 1:** Population diversity.

Population	*N*	*P_*N*_*	*H*_E_^1^	*H*_E_^2^	*A_S_*
Blacklip	65	0.846	0.330	0.350	0.771
Greenlip	87	0.846	0.343	0.364	0.752
Hybrid	39	0.778	0.335	0.386	0.793


*The number of animals (*N*), proportion of polymorphic loci (*P*_*N*_) and expected heterozygosity (*H*_*E*_) is given separately for each population. Expected heterozygosity was calculated by averaging all 105 SNP (*H*_*E*_^*1*^) or using only population specific polymorphic loci (*H*_*E*_^*2*^). The final diversity metric is averaged pairwise allele sharing (*A*_*S*_) calculated as identity by state. Increasing values indicate high similarity between individuals.*

### Population Divergence

**FIGURE 2:**
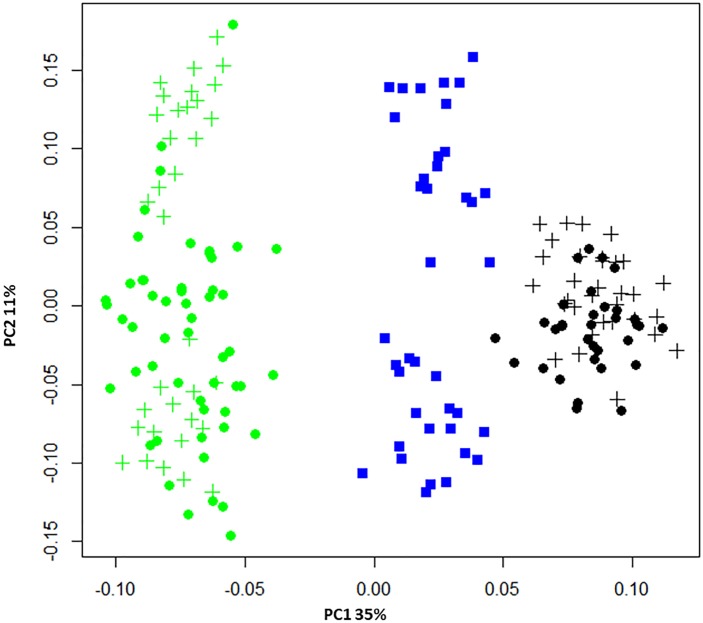
Principal component analysis (PCA) of pairwise genetic distance. The membership of populations is indicated using blue (hybrids), green (GL), or black (BL) symbols while for the two parentage species broodstock are denoted using circles and progeny using crosses. The proportion of variance captured is given as a percentage for both the first and second principal components (PC1 and PC2).

The power to discriminate between individuals and populations was assessed using PCA, a method that positions individuals without reference to their population of origin. Figure 2 plots the two largest principal components, revealing individuals from the same population were positioned in non-overlapping clusters. This confirmed the genotyping tool was able to distinguish between populations, despite its development employing a SNP prioritization approach which sought to maintain polymorphism across populations. Blacklip abalone appeared more tightly clustered, suggesting they are more closely related to each other than is the case for greenlip abalone which took more dispersed positions. Higher relatedness within BL compared with GL was also reflected in the observed *A*_S_ values (Table 1) and suggests a less diverse collection of broodstock.

**FIGURE 3:**
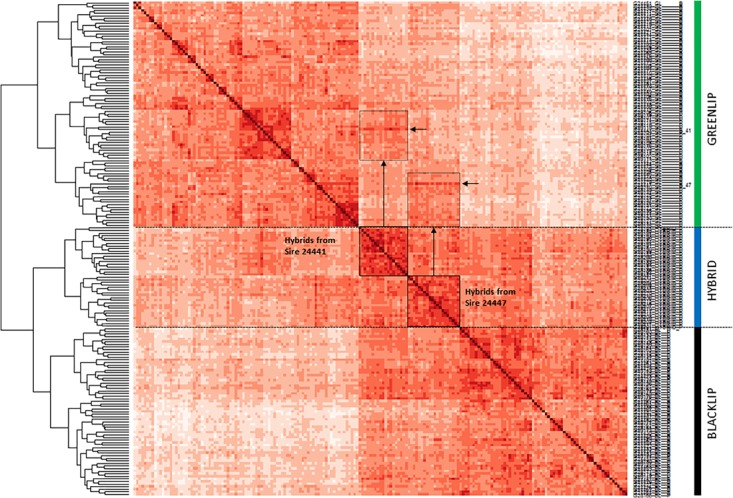
Relationship of 191 abalone from three populations. Pairwise allele sharing (*A*_S_) was used to construct a dendogram (at left) and heatmap. Cell color represents the strength of allele sharing, where increasingly dark shades indicate increasing relatedness between animals. Self-self comparisons appear on the diagonal with maximum *A*_S_ = 1 and darkest color. Hybrids branch as intermediate between the two parentage species, and consist of two groups which are indicated using boxes. Vertical arrows connect off diagonal regions of the heatmap with elevated *A*_S_ (hatched boxes). Rows corresponding to the greenlip sires used in the construction of the hybrids (sires 24441 and 24447) are shown with horizontal arrows.

A second approach sought to evaluate the relationship between individuals using SNP data, by using an allele sharing matrix to generate a dendogram and heatmap (Figure 3). As expected, hybrid abalone all took branch positions which were intermediate between the two parental species. The genotyping platform was also able to detect clear familial relationship blocks that correspond with the known pedigree. For example, inspection within the hybrid branch revealed two groups. The membership of each group corresponds exactly with half-sib individuals known to share a common greenlip sire. The off diagonal regions of the heatmap are able to link the greenlip sire responsible for generating the associated hybrid group (refer to the annotations on Figure 3). Taken together, this demonstrated the genotyping tool is able to reconstitute known relationships to the level of half-sib families.

### Parentage Analysis

Progeny in the Jade Tiger Abalone breeding program each have one known, and one unknown, true parent (Hamilton et al., 2009). Information describing known parent – progeny pairs allowed us to evaluate the ability of the SNP tool to accurately assign parentage using genotypic data. To assign paternity, 36 male broodstock were all made available for identification as the correct sire independent of population type (BL or GL). Similarly for the assignment of maternity, 50 female broodstock were all made available as candidate dams. The likelihood approach was used to identify the candidate sire and dam with highest *deltaLOD* for each progeny, before the assigned parent was matched against the true parent to estimate accuracy. The genotyping panel identified the true parent as the best candidate parent in each of the 105 parent – progeny pair tested, representing 100% accuracy. The average *deltaLOD* was similar where the true parent was known (9.63) or unknown (9.67, Table 2). This suggests the parent assigned using the SNP genotypic data alone is likely to be correct. The approach requires a genotype call in both progeny and parent, meaning a missing genotype in either renders the loci uninformative. The average number of SNP used to achieve 100% accuracy was less than 100, which is not the case in some parentage assignment scenarios (Strucken et al., 2017). This is likely the result of the high number of loci with high AFΔ in a breeding program incorporating multiple species which together generates strong discriminating power in a modest number of loci.

**Table 2:** Parentage assignment.

	*n*	True Parent	*deltaLOD*	
			
Progeny		Assignment	True Parent	Unknown Parent
Blacklip	32	100	7.67	12.84
Greenlip	34	100	12.57	8.30
Hybrid	39	100	8.58	8.44
Total	105		9.63	9.67


*Progeny and parents from three populations were used for parentage assignment using the likelihood approach implemented in Cervus v3.0.7. True parent assignment gives the percentage of progeny for which the best candidate parent matched the known true parent. Average *deltaLOD* values are given separately for assigned progeny – parent pairs where the true parent is known or unknown.*

## Discussion

A foundational element of applied breeding is the ability to collect trait measures on animals that have known parentage. In response we developed a SNP based genotyping tool capable of returning cost effective high quality genetic data. To begin, we designed the sequencing component of the project to ensure at least moderate genetic diversity was present within the animals used for SNP discovery. Skim sequencing 24 animals identified a high confidence set of 2.2 M SNP that represents a useful resource for the abalone research community. The number of animals used for SNP discovery is modest, but comparable to previous studies in aquaculture species (Palti et al., 2014). It is worthwhile noting that additional animals sourced from a wider genetic background would likely have identified a higher number of variants. None the less, the SNP identified are available for future development of high density SNP tool, or for design of single SNP assays that reside in candidate genes of interest in preparation for association analysis. We next used the SNP collection to identify loci displaying polymorphism in both parental species. This identified a surprisingly high rate of overlap, whereby nearly half of positions identified as SNP in one species were independently identified as polymorphic in the other. We interpret this high overlap (44%) as reflecting a low level of divergence between blacklip and greenlip abalone, which is consistent with the findings of a more comprehensive assessment of diversity within 14 *Haliotis* species distributed throughout the Indo-Pacific region (Degnan et al., 2006). Assessment of mtDNA sequence grouped *H. laevigata* and *H. rubra* tightly, even within the context of the six species of Australian temperate water abalone investigated. We did not attempt to use the sequence data to estimate divergence time given the shallow depth of coverage obtained per individual, however, a common karyotype (Botwright, 2015), production of fertile hybrids and a shared natural distribution in Australian waters all support our interpretation of recent divergence.

In preparation for construction of a low density SNP genotyping tool, we prioritized loci for assay design on the basis of allele frequency across populations. This sought to restrict assay design to loci that had been independently identified in BL, GL and HY pools with moderate allele frequency within each. The conversion of prioritized loci to working KASP assays was disappointingly low at 27%, with at least three possible explanations. We mapped sequence reads from the hybrid and BL pools against the greenlip reference to perform variant detection, possibly introducing some bias. Genotyping the validation populations showed no reduction in the proportion of polymorphic loci in the BL animals, suggesting our comparative mapping approach was not a strong factor in the low conversion rate. Alternatively, a more likely explanation may reside in the preliminary nature of the greenlip abalone reference genome that contains in excess of a hundred thousand contigs (Botwright et al., unpublished). This raises the possibility that complex repetitive structures such as CNV or segmental duplications have occurred that were not accounted for in our approach to identify prioritized SNP for assay design. A third possible explanation concerns undetected polymorphism immediately flanking assay SNP, however, we rate this as unlikely given i) genetic diversity was present in the panel of animals used for sequencing and SNP discovery and ii) the presence of flanking polymorphism was considered during SNP filtering.

Application of the SNP tool to genotype a validation population of 191 animals successfully demonstrated its ability to estimate population diversity and to accurately identify known pedigreed relationships. Analysis of pairwise genetic distance (Figure 2) suggests the tool has utility to assign samples of unknown origin to the correct population, and pairwise allele sharing revealed population substructure within each parental species and the hybrid population (Figure 3). It is worthwhile noting every animal tested had a unique genotypic complement, suggesting the tool is adequate to manage inbreeding in the context of an applied breeding program. Of particular relevance was evaluation of parentage assignment, which resulted in 100% accuracy in an analysis scenario that treated every available broodstock parent as a candidate. This is a more challenging analytical scenario than faced within the breeding program, where less than 10 candidate parents are used in any given crossing design (Hamilton et al., 2009). However, we note that the validation population was constructed using a limited number of sires and dams. This means that application of the SNP tool in a broader collection of families, and where very closely related broodstock have been employed, may be needed to more fully assess the power to accurately assign parentage. Our conclusion using the existing dataset is that application of 100 SNP appears to be sufficient to return highly accurate parentage assignment to support future genetic gain in this important aquaculture species.

## Ethics Statement

The DNA samples used are from the Jade Tiger Abalone breeding program, as part of the commercial operations of Jade Tiger Abalone Pty Ltd. Their use was in accordance with authorized management practices of the company.

## Author Contributions

JK conceived the experiments, performed the analysis, and wrote the manuscript. MH, SM, HK, and NB performed the analysis and drafted the manuscript. LM and AK contributed animal material and experimental design.

## Conflict of Interest Statement

The authors declare that the research was conducted in the absence of any commercial or financial relationships that could be construed as a potential conflict of interest.

## Footnotes

1 https://bitbucket.org/arobinson/quadtrim

2 https://github.com/samtools/bcftools
