# Supplementary material for: Genome Sequencing of Blacklip and Greenlip Abalone for Development and Validation of a SNP Based Genotyping Tool
Source: Front Genet. 2019 Jan 4;9:687. doi: 10.3389/fgene.2018.00687 (PMC6328477; doi:10.3389/fgene.2018.00687)
Supplement: Supplementary file 1 [file Table_1.docx]

**Supplementary Material**

**Table S1. SNP Pruning**

**Table S2. Read Mapping Statistics**

Blacklip (BL), Greenlip (GL) and Hybrid (HY) abalone sequence reads were each mapped to the greenlip abalone genome reference assembly. For each sequence dataset, the total number of trimmed reads is shown, along with the percentage successfully mapped. The mate pair architecture of library construction was exploited to determine the percentage of reads that mapped with their mate pair in the correct orientation and spacing. The last metric provided reflects the number of reads with a mate pair mapped to a different contig.
